# Studying the topology of peroxisomal acyl-CoA synthetases using self-assembling split sfGFP

**DOI:** 10.1007/s00418-023-02257-7

**Published:** 2024-01-19

**Authors:** Serhii Chornyi, Janet Koster, Lodewijk IJlst, Hans R. Waterham

**Affiliations:** 1 Department of Clinical Chemistry, Laboratory Genetic Metabolic Diseases, Amsterdam UMC-University of Amsterdam, Meibergdreef 9, 1105 AZ Amsterdam, The Netherlands; 2 Amsterdam Gastroenterology Endocrinology Metabolism, Amsterdam, The Netherlands; 3 Amsterdam Reproduction and Development, Amsterdam, The Netherlands

**Keywords:** Superfolder GFP, Fatty acids, CoA, Metabolism, Peroxisomes

## Abstract

Peroxisomes are membrane-bounded organelles that contain enzymes involved in multiple lipid metabolic pathways. Several of these pathways require (re-)activation of fatty acids to coenzyme A (CoA) esters by acyl-CoA synthetases, which may take place inside the peroxisomal lumen or extraperoxisomal. The acyl-CoA synthetases SLC27A2, SLC27A4, ACSL1, and ACSL4 have different but overlapping substrate specificities and were previously reported to be localized in the peroxisomal membrane in addition to other subcellular locations. However, it has remained unclear if the catalytic acyl-CoA synthetase sites of these enzymes are facing the peroxisomal lumen or the cytosolic side of the peroxisomal membrane. To study this topology in cellulo we have developed a microscopy-based method that uses the previously developed self-assembling split superfolder (sf) green fluorescent protein (GFP) assay. We show that this self-assembling split sfGFP method can be used to study the localization as well as the topology of membrane proteins in the peroxisomal membrane, but that it is less suited to study the location of soluble peroxisomal proteins. With the method we could demonstrate that the acyl-CoA synthetase domains of the peroxisome-bound acyl-CoA synthetases SLC27A2 and SLC27A4 are oriented toward the peroxisomal lumen and the domain of ACSL1 toward the cytosol. In contrast to previous reports, ACSL4 was not found in peroxisomes.

**Supplementary Information:**

The online version contains supplementary material available at 10.1007/s00418-023-02257-7.

## Introduction

Peroxisomes are metabolic organelles that enclose multiple enzymes and metabolic pathways (Wanders et al. 2023), which are required, among others, for the degradation of straight-chain fatty acids, via peroxisomal β-oxidation, branched-chain fatty acids, α- and β-oxidation, and the synthesis of ether phospholipids. To become substrate for the peroxisomal pathways, the fatty acids have to become activated to CoA esters to allow: (1) their ABCD-mediated import into peroxisomes, (2) their intraperoxisomal degradation, and (3) their involvement in de novo ether phospholipid synthesis. The activation of fatty acids to CoA esters is catalyzed by membrane-associated acyl-CoA synthetases, of which multiple are found in human cells and often at different subcellular locations. Four of these have been reported to be (co-)localized to peroxisomal membranes, although their topologies in the peroxisomal membrane have not been definitely resolved (Watkins 2008; Watkins and Ellis 2012).

To become a substrate for peroxisomal lipid metabolism, saturated, mono- and polyunsaturated very long-chain, long-chain, dicarboxylic, and branched-chain fatty acids are first activated in the cytosol to their corresponding acyl-CoA esters and subsequently imported into peroxisomes by one or more of the three known peroxisomal transporter proteins that belong to the ATP binding cassette transporters subfamily D (ABCD) (Kemp et al. 2011; van Roermund et al. 2014). It has been shown that the peroxisomal ABCD transporters in yeasts, plants, and humans have thioesterase activity that is a requirement for the import and most probably results in the hydrolysis of the acyl-CoAs during transport, after which the free fatty acids and CoA are imported into peroxisomes separately. To become a substrate for the intraperoxisomal degradation via β- or α-oxidation, the free fatty acids have to be reconverted to acyl-CoA esters inside the peroxisomal lumen (van Roermund et al. 2012; De Marcos Lousa et al. 2013; Carrier et al. 2019; Kawaguchi et al. 2021). This reactivation of the acyl-CoA esters after the ABCD-mediated import into peroxisomes is probably performed by one or more peroxisomal acyl-CoA synthetases.

Branched-chain fatty acids, such as phytanic acid, are degraded inside peroxisomes via α- and β-oxidation. Through peroxisomal α-oxidation, the CoA ester of phytanic acid is converted to pristanic acid, which then needs to be activated to pristanoyl-CoA to become a substrate of subsequent peroxisomal β-oxidation; this activation is probably performed by a peroxisomal acyl-CoA synthetase (Wanders et al. 2011; Watkins and Ellis 2012).

The first steps of de novo synthesis of ether lipids are mediated by the peroxisomal lumen enzymes glyceronephosphate O-acyltransferase (GNPAT) and alkylglycerone phosphate synthase (AGPS). GNPAT transfers the long-chain fatty acid from acyl-CoA to dihydroxyacetone phosphate (DHAP), forming acyl-DHAP, while AGPS replaces the fatty acid with a long-chain alcohol, thus forming alkyl-DHAP and a free fatty acid (Zomer et al. 1993). The alkyl-DHAP is subsequently used as a precursor for the synthesis of plasmalogens and other ether lipids (Nagan and Zoeller 2001). We have recently shown that the long-chain acyl-CoAs required for GNPAT are imported from the cytosol into peroxisomes by the ABCD transporters or generated during the β-oxidation-mediated shortening of very long-chain acyl-CoAs inside the peroxisomes (Chornyi et al. 2023). However, it remained unclear if the free fatty acid released by AGPS during alkyl-DHAP synthesis is exported from peroxisomes or can be reactivated to a CoA ester inside the peroxisomal lumen and then reused by the GNPAT enzyme.

So far, four different acyl-CoA synthetases with distinct but overlapping substrate specificities have been reported in peroxisomes in mammalian cells—SLC27A2 (also known as FATP2 and ACSVL1), SLC27A4 (also known as FATP4, ACSVL5, and ACSVL4), ACSL1, and ACSL4 (Uchiyama et al. 1996; Lewin et al. 2002; Kikuchi et al. 2004; Wiese et al. 2007; Gronemeyer et al. 2013). These acyl-CoA synthetases all have predicted transmembrane domain(s) and were found to be associated with peroxisomal membranes. Based on enzyme activity measurement-based latency studies of isolated peroxisomes treated with proteases, it was concluded that the acyl-CoA synthetase activity domains of the enzymes are exposed to the cytosol (Mannaerts et al. 1982; Lageweg et al. 1991; Pahan and Singh 1995). However, this does not correspond with the above discussed required intraperoxisomal roles of the synthetases in peroxisomal metabolism. Moreover, using antibodies raised against the N- or C-terminal peptides of SLC27A2, Smith et al. provided evidence that SLC27A2 is facing the peroxisomal lumen (Smith et al. 2000). Also, in yeast (Hettema et al. 1996) and plants (Fulda et al. 2004) acyl-CoA synthetase activities were found inside the peroxisomal lumen. To resolve this discrepancy, we developed a method to study the topology of peroxisomal membrane proteins in cellulo and used this method to determine the topology of the acyl-CoA synthetases in the peroxisomal membrane of human HeLa cells.

We made use of the self-assembling split superfolder GFP (sfGFP) approach (Cabantous et al. 2005). This approach uses a bipartite self-assembling split sfGFP construct that consists of two nonfluorescent parts of sfGFP, GFP(1–10) and GFP11, with a self-complementation ability to form a complete fluorescent protein. In our method, the protein of interest is tagged at its N- or C-terminus with GFP11 and then coexpressed with GFP(1–10) targeted to either the cytosol or the peroxisomal lumen, the latter by extending the GFP(1–10) protein with a C-terminal peroxisomal targeting signal type 1 [GFP(1–10)-PTS1]. When GFP11 and GFP(1–10) are in close proximity, which requires the location in the same compartment, they will physically interact and acquire green fluorescence properties, which can be assessed with fluorescence microscopy. Using this method, we showed that the coexpression of an N- or C-terminal GFP11-tagged peroxisomal membrane protein with cytosolic GFP(1–10) or peroxisomal GFP(1–10)-PTS1 allows for confirmation of its subcellular location and resolution of its topology. With the method, we showed that peroxisomal acyl-CoA synthetases SLC27A2 and SLC27A4 are oriented in the peroxisomal membrane with their acyl-CoA synthetase domains facing the peroxisomal lumen; however, the acyl-CoA synthetase domain of ACSL1 faces the cytosol.

## Materials and methods

### Cell culture

HeLa cells were routinely cultured at 37 °C under an atmosphere of 5% CO_2_ in Dulbecco’s modified Eagle’s medium (DMEM, high glucose, Gibco) supplemented with 10% fetal bovine serum (Capricorn Scientific), 25 mM HEPES buffer (VWR), and antibiotics [100 U/mL penicillin (Gibco), 100 μg/mL streptomycin (Gibco), and 250 ng/mL amphotericin B (Gibco)].

### Molecular biological techniques

Plasmids were constructed and amplified in *Escherichia coli* DH5α and purified using a QIAGEN Plasmid Midiprep kit (QIAGEN) according to the manufacturer’s instructions. Plasmids were made using standard restriction digestion–ligation-based methods in *E. coli*, using Advantage 2 Polymerase Mix (Takara Bio), T4 DNA ligase, restriction enzymes, oligonucleotides, and buffers supplied by New England Biolabs. Relevant DNA sequences of newly made plasmids were confirmed by Sanger Sequencing, which was carried out by the Core Facility Genomics of Amsterdam UMC, and the results were analyzed using CodonCode Aligner (version 8.0.2).

A plasmid encoding GFP(1–10) was obtained via Addgene from Bo Huang [pcDNA3.1-GFP(1–10); Addgene plasmid no. 70219 (Kamiyama et al. 2016)]. To generate GFP(1–10)-PTS1 (targeted to peroxisomes), the coding sequence of GFP(1–10) was subcloned without a stop codon into the pcDNA5/frt plasmid (Invitrogen) and extended with two complementary oligonucleotide primers encoding the carboxy terminus of ACOX3, including its peroxisomal targeting signal (PTS1), preceded by a linker GSGGE (GSGGENKPVIGSLKSKL*) (for primers see Table S1).

To tag proteins with GFP11 at the N- or C-terminus, we constructed plasmids that contain an ATG-*GFP11* short-linker sequence followed by a multiple cloning site (plasmid *GFP11*-C-pcDNA3) or a multiple cloning site followed by short linker*-GFP11*-stop codon (plasmid N-*GFP11*-pcDNA3) using the pcDNA3 plasmid (Invitrogen) and primers indicated in Table S1. The coding sequences of selected proteins were PCR-amplified with or without a stop codon and respectively cloned into the *GFP11*-C-pcDNA3 plasmid to generate N-terminally GFP11-tagged variants or into the N-*GFP11*-pcDNA3 plasmid to generate C-terminally GFP11-tagged variants. The coding sequences of *SLC27A2* (NM_003645.4) and *SLC27A4* (NM_005094.4) were PCR-amplified from plasmids provided by Yusuke Ohno (Ohkuni et al. 2013), and the coding sequences of *ABCD1* (NM_000033.4) were amplified from the pLB741 plasmid (Smith et al. 1999). The coding sequences of *ACOX1* (NM_004035.7), *ACSL1* (NM_001286708.2), and *ACSL4* (NM_022977.3) were PCR-amplified from human complementary DNA (cDNA), using the primers indicated in Table S1. For this, total cDNA was prepared from RNA isolated from HEK293 cells by trizol extraction (Sigma-Aldrich) using the QuantiTect Reverse Transcription kit (QIAGEN). A site-directed mutagenesis kit (NEB, E0554S) was used to remove the stop codon from the coding sequence of *ACOX1* after it was cloned into N-GFP11-pcDNA3. The coding sequences of *GPI* (NM_001289789.1) and *SLC25A17* (NM_006358.4) with an GFP11 tag at the N- or C-terminus were synthesized by GenScript Biotech and subcloned into the pcDNA3.1 plasmid.

### Cell transfection

Cells were seeded in 6-well plates (40–60% confluency, 2 mL of culture medium per well) and transfected using jetPRIME according to the manufacturer’s protocol (Polyplus). For transfection of one plasmid we used 2 µg of DNA and of two plasmids we used 1 µg of each plasmid. For colocalization with mApple-PTS1, we added an additional 0.3 µg plasmid encoding mApple-PTS1. Plasmids were mixed with 4 µL of jetPRIME reagent and 200 µL of jetPRIME buffer, and the solution was added to the cells in the 6-well plate. Culture medium was refreshed after overnight incubation. A total of 24–48 h after transfection (unless specified otherwise), cells were plated on coverslips for live-cell imaging (Ibidi, µ-Slide 4 well glass bottom) or on regular coverslips for immunofluorescence analysis (Epredia, 21 × 26 mm no. 1) and allowed to adhere by culturing overnight at 37 °C under an atmosphere of 5% CO_2_. For live-cell imaging, cells were incubated in phenol red-free DMEM high glucose (Gibco) supplemented with 10% fetal bovine serum (Capricorn Scientific).

Mitochondria were stained with Mitotracker Red 580 (Molecular Probes, M22425; 200 nM dissolved in culture medium with 30 min incubation). The endoplasmic reticulum (ER) was stained with ER tracker blue-white DPX (Molecular Probes, E12353; 0.5 µM dissolved in culture medium with 10 min incubation).

### Immunofluorescence analysis

Cells were washed with phosphate-buffered saline (PBS) and fixed by incubation with 2% paraformaldehyde for 15 min. Next, membranes were permeabilized by incubation with PBS solution containing 0.1% (v/v) Triton X-100 (Bio-Rad, 1610407) for 5 min, and the coverslips were blocked using 10 g/L bovine serum albumin dissolved in PBS for 1 h. Primary and secondary antibodies were diluted in 10 g/L bovine serum albumin in PBS and used for incubation for 1 h at room temperature. We used primary antibodies against GFP (Santa Cruz, sc-8334; 1:1000) or ACBD5 (Sigma, HPA012145; 1:500) and secondary antibodies anti-rabbit IgG Alexa 594 (Invitrogen, A-11012; 1:500) or sequentially biotinylated anti-rabbit IgG (DAKO, E0432; 1:200) and a streptavidin–fluorescein isothiocyanate (FITC) complex (DAKO, 11-4317-87; 1:200). The glass coverslips were fixed on objective slides with the mounting medium ProLong^TM^ Gold anti-fade reagent with DAPI (Invitrogen, P36935).

### Microscopy imaging

Live-cell and immunofluorescence imaging were performed using a Leica TCS SP8 SMD confocal microscope with a full-case incubator at 37 °C under an atmosphere of 5% CO_2_ using LASX software (version 3.5.7.23225). The objective lens was HC PL APO CS2 63×/1.40 oil. Fluorescence images were captured in a sequential scan mode with a scan speed 100 or 10 Hz and a line average of 6 and airy units 1.0, using 1024 × 1024 pixels.

The live-cell imaging settings were as follows: sfGFP (green images) was excited with a Leica white light laser (488 nm) and detected with a HyD Leica detector (498–530 nm); mApple-PTS1 (red images) was excited with a Leica white light laser (540 nm) and detected with a HyD Leica detector (600–650 nm); MitoTracker (red images) was excited with a Leica white light laser (594 nm) and detected with a HyD Leica detector (600–660 nm); ER tracker (red images) was excited with a Leica ultraviolet (UV) laser (405 nm) and detected with a HyD Leica detector (410–470 nm). Immunofluorescence imaging settings were as follows: DAPI (blue images) was excited with a Leica UV laser (405 nm) and detected with a PMT Leica detector (418–460 nm); FITC (green images) was excited with a Leica white light laser (488 nm) and detected with a HyD Leica detector (500–550 nm); Alexa594 (red images) was excited with a Leica white light laser (594 nm) and detected with a HyD detector (600–640 nm).

All live-cell or immunofluorescence experiments were repeated four to six independent times.

Contrast enhancement and an intensity threshold were used to exclude the background and for optimal image presentation. Image adjustments were performed identically for all images in the same experiment with LASX 3D software (version 3.7.1). Manuscript figures were prepared using Adobe Illustrator software.

### Protein structure predictions

Protein structure predictions were obtained from AlphaFold, and the presented protein structure images are false-colored according to the per-residue confidence score of the protein structure [https://alphafold.ebi.ac.uk/ (Jumper et al. 2021; Varadi et al. 2022)]. Prediction of transmembrane helices in proteins was done using the DeepTMHMM server [https://dtu.biolib.com/DeepTMHMM (Hallgren et al. 2022)] and the TMHMM server [https://services.healthtech.dtu.dk/services/TMHMM-2.0/ (Sonnhammer et al. 1998)].

## Results

### GFP11 tag allows for studying the topology of peroxisomal membrane proteins

**Fig. 1:**
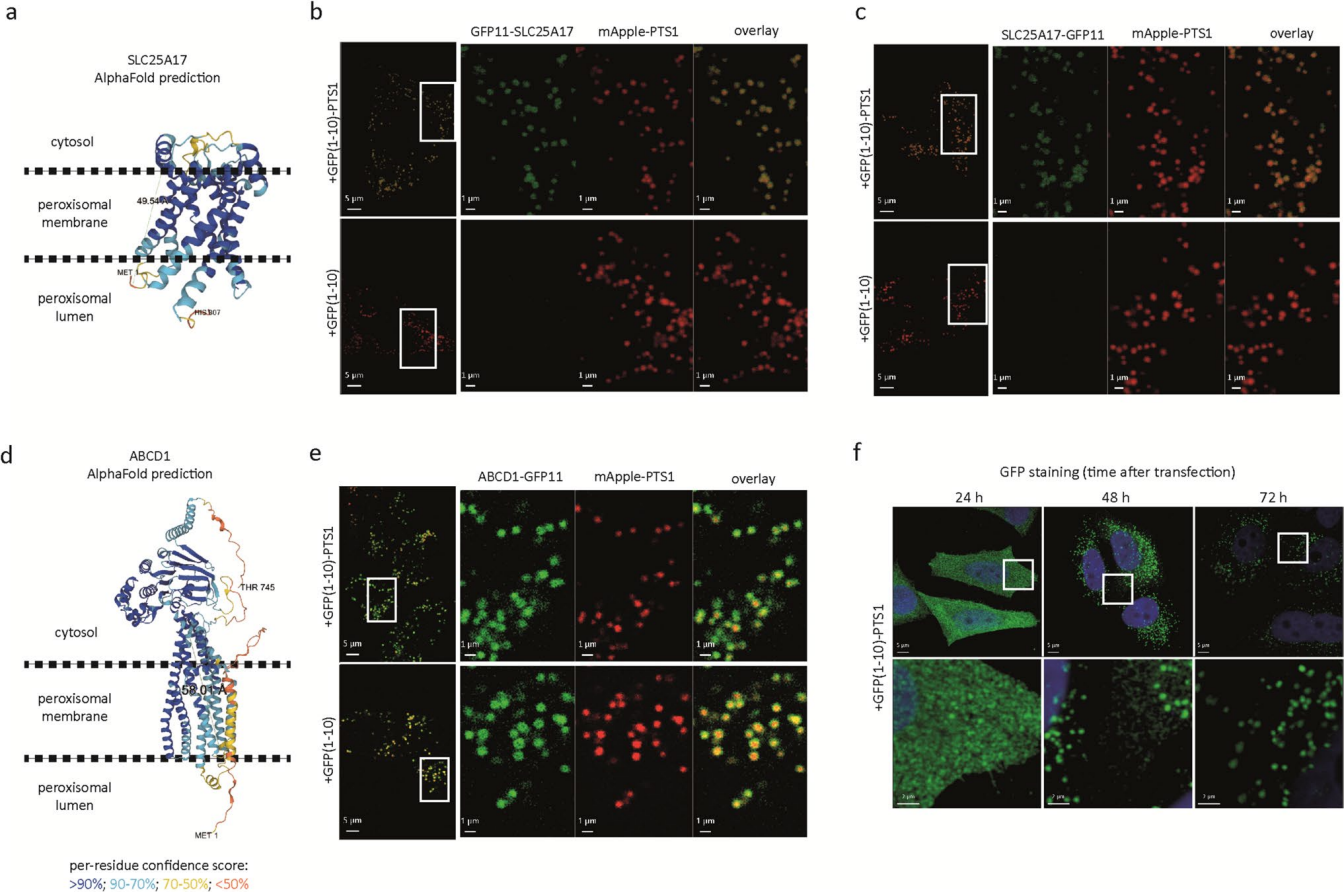
The GFP11 tag allows for studying the topology of peroxisomal membrane proteins. **a** and **d** AlphaFold structure prediction of peroxisomal membrane proteins SLC25A17 and ABCD1. The orientation of the proteins in the peroxisomal membrane is schematically depicted. **b**, **c**, and **e** Live-cell imaging of HeLa cells cotransfected with three plasmids encoding for peroxisomal lumen marker mApple-PTS1, GFP(1–10) or GFP(1–10)-PTS1, and **b** GFP11-SLC25A17, **c** SLC25A17-GFP11, or **e** ABCD1-GFP11. The sfGFP signal appears as green and mApple signal as red; images at the right side represent overlays of sfGFP and mApple-PTS1. **f** Immunofluorescence analysis of cells transfected with a plasmid encoding GFP(1–10)-PTS1 24, 48, or 72 h after transfection using anti-GFP antibodies (FITC, green). DAPI signal appears as blue; the images are presented as overlay of DAPI and sfGFP

Expression of GFP(1–10) in cells results in a cytosolic localization. To specifically target GFP(1–10) to the peroxisomal lumen, we added the coding sequence of a type 1 peroxisomal targeting signal to its C terminus [GFP(1–10)-PTS1]. To verify that GFP(1–10)-PTS1 localizes to the peroxisomal lumen and can interact with GFP11 when used as a N- or C-terminal tag of peroxisomal membrane proteins, we examined the topology of peroxisomal membrane protein SLC25A17, also known as PMP34. Based on the predicted protein structure (Fig. 1a), both the C- and N-terminal ends of SLC25A17 face the same side of the membrane. Although, as far as we know, the topology of the SLC25A17 has not been reported previously, we expected both the C- and N-terminal ends to face the peroxisomal lumen, similar to *Sc*Ant1p, the close ortholog of SLC25A17 in the yeast *Saccharomyces cerevisiae* (van Roermund et al. 2022). We coexpressed SLC25A17, tagged with GFP11 at the N- or C- terminal end, with GFP(1–10) or GFP(1–10)-PTS1 and mApple-PTS1 in HeLa cells. Coexpression of both GFP11-tagged SLC25A17 proteins with GFP(1–10)-PTS1 resulted in a clear punctuated sfGFP fluorescence signal, which colocalized with the artificial peroxisomal lumen marker mApple-PTS1 (Fig. 1b, c). In contrast, no sfGFP fluorescence signal was observed when the GFP11-tagged SLC25A17 proteins were coexpressed with cytosolic GFP(1–10) (Fig. 1b, c). These results show that GFP11 attached to either the N- or the C-terminal end of SLC25A17 can efficiently interact with peroxisomal GFP(1–10)-PTS1 to generate the complete fluorescent protein and confirm that, similar to *Sc*Ant1p, the C- and N-terminal ends of SLC25A17 face the peroxisomal lumen.

To verify that cytosolic GFP(1–10) can interact with GFP11-tagged peroxisomal membrane proteins exposing the GFP11 tag at the cytosolic side of the peroxisomal membrane, we examined the topology of another known peroxisomal membrane protein, ABCD1, the C-terminal end of which was shown previously to face the cytosolic side of the peroxisomal membrane [(Le et al. 2022), see also Fig. 1d]. When we coexpressed ABCD1 C-terminally tagged with GFP11 (ABCD1-GFP11) with cytosolic GFP(1–10) and the peroxisomal lumen marker mApple-PTS1, we observed a punctuated sfGFP fluorescence signal that colocalized with mApple-PTS1 (Fig. 1e) confirming that the GFP11 tag in combination with GFP(1–10) also allows for studying the topology of peroxisomal membrane proteins when facing the cytosol.

Unexpectedly, when we coexpressed ABCD1-GFP11 with GFP(1–10)-PTS1, we also observed a punctated sfGFP fluorescence signal that colocalized with mApple-PTS1 (Fig. 1e). At first sight, this seemed to suggest that the C-terminus of ACBD1-GFP11 can also be located in the peroxisomal lumen. However, given that it has been shown that proteins destined for peroxisomes, thus also GFP(1–10)-PTS1, are synthesized on free ribosomes in the cytosol and only imported into peroxisomes after their synthesis (Van Ael and Fransen 2006), we hypothesized that due to the high-affinity interaction between GFP(1–10) and GFP11 (Cabantous et al. 2005; Pédelacq et al. 2006), GFP(1–10)-PTS1 already interacts in the cytosol with the GFP11 tag before it is imported into peroxisomes, thus forming a stable fluorescent complex at the cytosolic face of the peroxisomal membrane. To find support for this, we studied the subcellular localization of GFP(1–10)-PTS1 in transfected cells by immunofluorescence microscopy using anti-GFP antibodies. This revealed that 24 h after the transfection, GFP(1–10)-PTS1 was still predominantly localized in the cytosol of virtually all cells (Fig. 1f). However, 48 and 72 h after transfection, GFP(1–10)-PTS1 was localized exclusively to peroxisomes in the majority of cells (Fig. 1f). This thus indicates that in the first 24 h after transfection, the rate of GFP(1–10)-PTS1 protein synthesis in the transfected cells is higher than the peroxisomal protein import rate, thus causing an accumulation of GFP(1–10)-PTS1 in the cytosol where it can interact with cytosolic GFP11. Of note, mCherry-PTS1, as well as other PTS1-targeted fluorescent proteins, is also localized primarily in the cytosol 24 h after the transfection (data not shown).

Based on these findings, we conclude that the self-assembling split sfGFP method can be used to study the topology of peroxisomal membrane proteins. However, because GFP(1–10)-PTS1 can transiently accumulate in the cytosol where it can interact with cytosolic GFP11, a positive fluorescent signal obtained upon coexpression of a GFP11-tagged membrane protein with GFP(1–10)-PTS1 can only be interpreted correctly when the GFP11-tagged membrane protein is also coexpressed with GFP(1–10).

### GFP(1–10)-PTS1 can coimport GFP11-tagged nonperoxisomal proteins into peroxisomes (piggybacking)

**Fig. 2:**
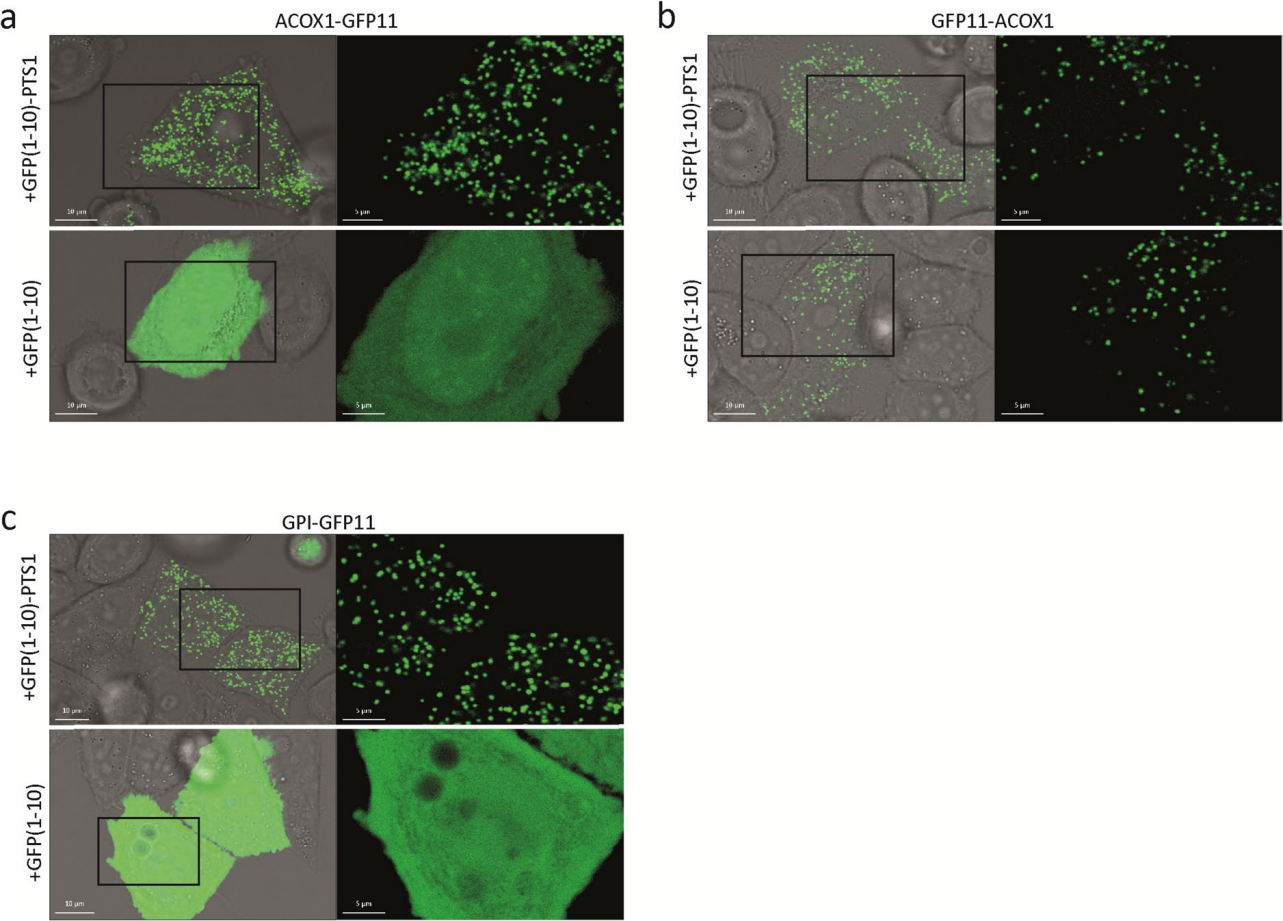
Soluble proteins are coimported into peroxisomes from the cytosol in complex with GFP(1–10) or GFP(1–10)-PTS1 when one of the proteins contains a PTS1 signal. Live-cell imaging of HeLa cells cotransfected with two plasmids encoding GFP(1–10) or GFP(1–10)-PTS1 and **a** ACOX1-GFP11, **b** GFP11-ACOX1, or **c** GPI-GFP11. The sfGFP signal appears as green and brightfield appears as gray; the image on the left side is the overlay of sfGFP and brightfield

In addition to studying the topology of peroxisomal membrane proteins, we also explored whether the same approach can be used to demonstrate the peroxisomal localization of soluble proteins. To this end, we tagged the known peroxisomal lumen protein acyl-CoA oxidase 1 (ACOX1) with GFP11 at its N- or C-terminus and coexpressed these with either GFP(1–10) or GFP(1–10)-PTS1. ACOX1 contains a C-terminal PTS1 that is recognized by the cytosolic peroxisomal lumen protein receptor PEX5, which targets the protein to the peroxisome. The addition of GFP11 to the C-terminus of ACOX1 is expected to block the interaction with PEX5 and thus prevent peroxisomal import, whereas the N-terminal GFP11-tagged ACOX1 should still be recognized and imported. When coexpressed with GFP(1–10)-PTS1, however, not only N- but also C-terminal GFP11-tagged ACOX1 showed a punctated sfGFP fluorescence signal indicating a peroxisomal location (Fig. 2a, b). Moreover, when coexpressed with GFP(1–10), N-terminal GFP11-tagged ACOX1 still showed a punctated sfGFP fluorescence signal, while C-terminal GFP11-tagged ACOX1 showed a diffuse, cytosolic sfGFP fluorescence signal (Fig. 2a, b). These findings can be explained by the previously reported and well-studied phenomena that (1) peroxisomal lumen proteins can assemble as protein complexes in the cytosol that are subsequently imported as a complex into peroxisomes (reviewed in Lanyon-Hogg et al. 2010), and (2) proteins lacking a peroxisomal targeting signal (PTS) can be coimported into peroxisomes through interaction with a PTS-containing protein, a mechanism also known as piggybacking (reviewed in Léon et al. 2006). Thus, due to the strong interaction in the cytosol of GFP(1–10) with the GFP11 tag, C-terminal GFP11-tagged ACOX1 is coimported with GFP(1–10)-PTS1, and GFP(1–10) is coimported with N-terminal GFP11-tagged ACOX1, explaining the peroxisomal sfGFP fluorescence signal in these two conditions. When GFP(1–10) is coexpressed with C-terminal GFP11-tagged ACOX1, however, peroxisomal coimport cannot occur, as this complex is not recognized by PEX5, resulting in a cytosolic sfGFP fluorescence signal.

To find further support for this explanation, we coexpressed a GFP11-tagged cytosolic protein glucose-6-phosphate isomerase (GPI) with GFP(1–10) or GFP(1–10)-PTS1. As expected, coexpression of C-terminally GFP11-tagged GPI with GFP(1–10) resulted in a cytosolic sfGFP fluorescence signal (Fig. 2c). However, when we coexpressed C-terminally GFP11-tagged GPI with GFP(1–10)-PTS1, we observed a punctated sfGFP fluorescence signal (Fig. 2c) that colocalized with the peroxisomal marker ACBD5 (Figure S1a), confirming that the GFP11-tagged cytosolic GPI protein can be coimported via piggybacking from the cytosol into peroxisomes through interaction with GFP(1–10)-PTS1.

These findings imply that the self-assembling split sfGFP assay is less suited to study the peroxisomal localization of soluble proteins and should be used with caution.

### The acyl-CoA synthetase domains of the peroxisomal membrane proteins SLC27A2 and SLC27A4 are facing the peroxisomal lumen and the domains of ACSL1 are facing the cytosol

**Fig. 3:**
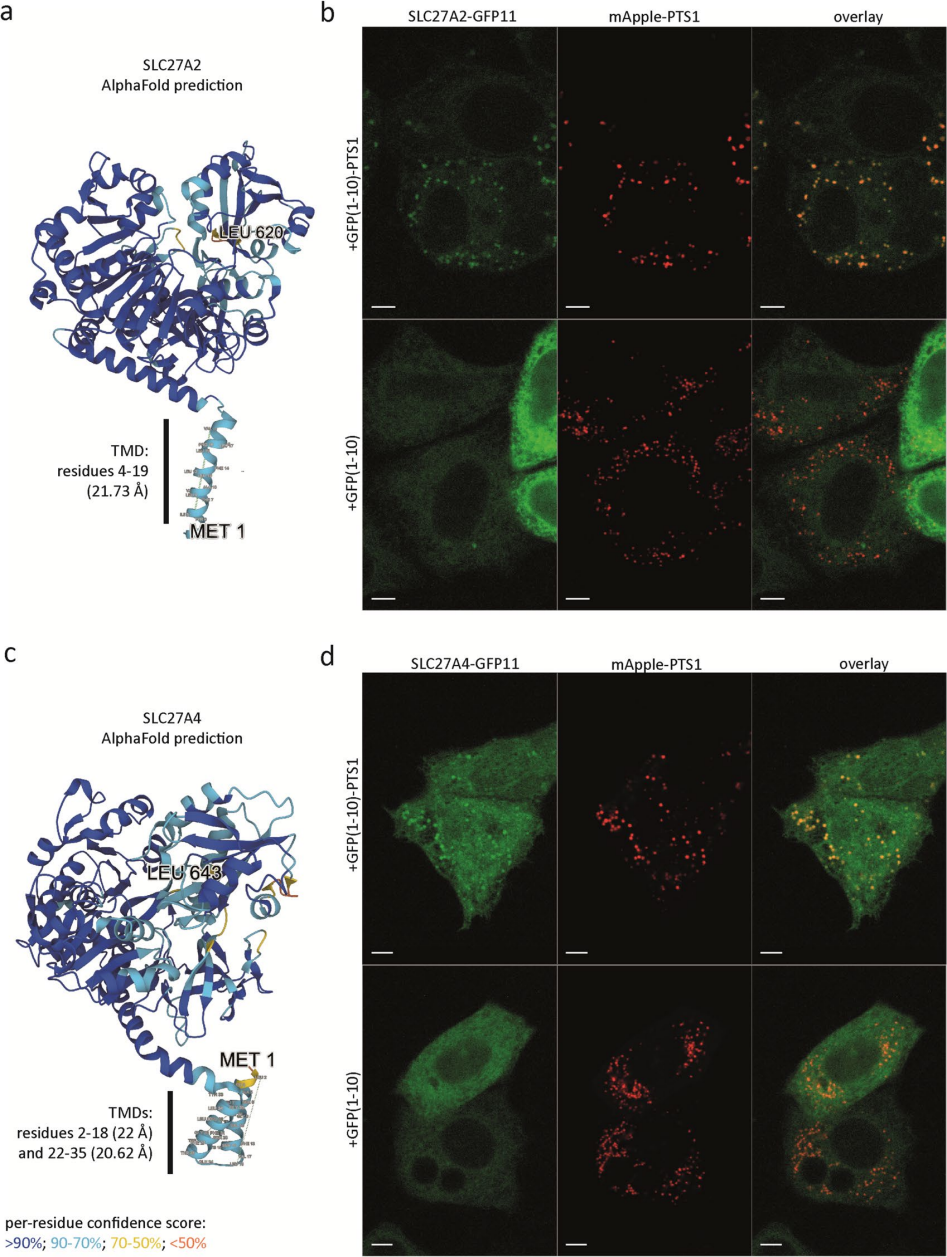
The acyl-CoA synthetase domains of SLC27A2 and SLC27A4 are facing the peroxisomal lumen. **a** and **c** AlphaFold structure prediction of membrane proteins SLC27A2 and SLC27A4 (TMD, predicted transmembrane domain). **b** and **d** Live-cell imaging of HeLa cells cotransfected with three plasmids encoding for peroxisomal lumen marker mApple-PTS1, GFP(1–10) or GFP(1–10)-PTS1, and **b** SLC27A2-GFP11 or **d** SLC27A4-GFP11. The sfGFP signal appears as green and mApple signal appears as red; the images at the right side represent overlays of sfGFP and mApple-PTS1; scale bar, 5 µm

**Fig. 4:**
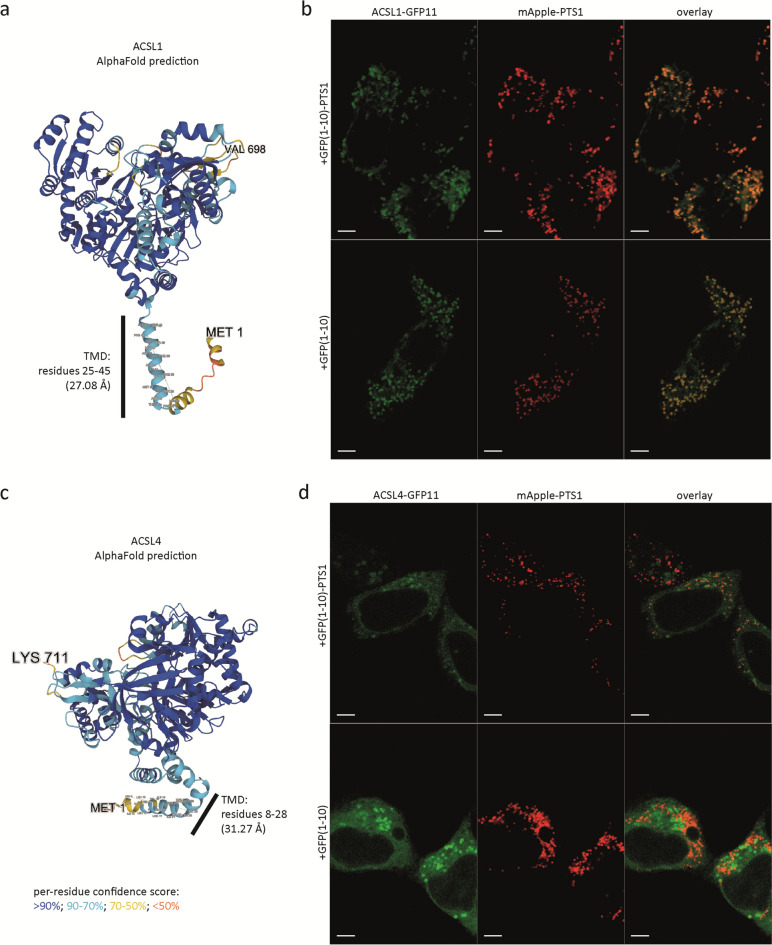
The acyl-CoA synthetase domain of ACSL1 is facing the cytosolic side of the peroxisomal membrane, but ACSL4 is not localized to peroxisomes in HeLa cells. **a** and **c** AlphaFold structure prediction of membrane proteins ACSL1 and ACSL4 (TMD, predicted transmembrane domain). **b** and **d** Live-cell imaging of HeLa cells cotransfected with three plasmids encoding for peroxisomal lumen marker mApple-PTS1, GFP(1–10) or GFP(1–10)-PTS1, and **b** ACSL1-GFP11 or **d** ACSL4-GFP11. The sfGFP signal appears as green and mApple signal appears as red; the images at the right side represent overlays of sfGFP and mApple-PTS1; scale bar, 5 µm

We used the above-described self-assembling split sfGFP method to confirm the location and study the topology of the acyl-CoA synthetase domains of the membrane-associated acyl-CoA synthetases SLC27A2, SLC27A4, ACSL1, and ACSL4 in the peroxisomal membrane. Previous studies reported that these proteins have different subcellular locations, including mitochondrial, ER, lipid droplets, and/or a peroxisomal membranes (Lewin et al. 2001; Milger et al. 2006; Jia et al. 2007; Ohkuni et al. 2013; Küch et al. 2014; Young et al. 2018). Structural prediction analysis indicates that all four synthetases have transmembrane domains near their N-terminus and that the acyl-CoA synthetase domains face the same side of the membrane as their C-terminus (Fig. 3, 4). To confirm the peroxisomal location of the four synthetases in HeLa cells and determine the topology of their acyl-CoA synthetase domains in the peroxisomal membrane, we therefore tagged their C termini with GFP11 and coexpressed each tagged protein with GFP(1–10) or GFP(1–10)-PTS1.

When we coexpressed C-terminal GFP11-tagged SLC27A2 or SLC27A4 with GFP(1–10), we observed an sfGFP fluorescence signal that looked similar to ER staining (Fig. 3b, d, S1b, and c), which is in accordance with the previously reported location of the proteins in the ER (Steinberg et al. 1999; Milger et al. 2006; Jia et al. 2007; Ohkuni et al. 2013). However, when C-terminal GFP11-tagged SLC27A2 or SLC27A4 was coexpressed with GFP(1–10)-PTS1 and the peroxisomal lumen marker mApple-PTS1, we observed an sfGFP fluorescence signal that colocalized with mApple-PTS1, indicating that the acyl-CoA synthetase domains of SLC27A2 and SLC27A4 face the peroxisomal lumen. In line with the previously reported topology of SLC27A4 in the ER membrane (Milger et al. 2006), we also observed some ER staining upon coexpression with GFP(1–10)-PTS1, which is most probably due to the above described observation that GFP(1–10)-PTS1 transiently accumulates in the cytosol and then can interact with GFP11 exposed to the cytosol.

When we coexpressed C-terminal GFP11-tagged ACSL1 with GFP(1–10)-PTS1 and mApple-PTS1, we found an sfGFP fluorescence signal that partially colocalized with mApple-PTS1 (Fig. 4b) but also with mitochondria (Figure S1f), indicating a partial peroxisomal location, in line with previous reports (Wiese et al. 2007; Gronemeyer et al. 2013; Young et al. 2018). The observed mitochondrial sfGFP signal is in line with the reported localization and topology of ACSL1 in the outer mitochondrial membrane (reviewed by Coleman 2019). When we coexpressed the GFP11-tagged ACSL1 with cytosolic GFP(1–10), we also observed sfGFP fluorescence, which colocalized with mApple-PTS1 and mitochondria (Fig. 4b, S1f), implying that, similar as described above for ABCD1 (Fig. 1e), the C-terminus of ACSL1, which includes the acyl-CoA synthetase domain, is facing the cytosolic side of the peroxisomal membrane.

Coexpression of C-terminal GFP11-tagged ACSL4 with GFP(1–10) or GFP(1–10)-PTS1 confirmed the previously reported location of the protein in lipid droplets and probably the ER (Fig. 4d) (Küch et al. 2014). However, we did not observe any peroxisomal staining when it was coexpressed with GFP(1–10)-PTS1 and, thus, could not confirm previous reports that indicated that this protein is also located in peroxisomes.

## Discussion

Peroxisomes harbor a large number of enzymes involved in multiple metabolic pathways often shared with other cellular compartments, including mitochondria, the ER, and the cytosol. While this requires an efficient exchange of substrates, products and cofactors between the compartments for which some specific peroxisomal membrane-bound transporter proteins have been identified, it remains unclear how some of these metabolites are transported and thus additional membrane proteins are likely to be identified (Chornyi et al. 2021). In addition to membrane proteins with an exclusive peroxisomal localization, peroxisomes have been found to share membrane proteins with other subcellular compartments (Yifrach et al. 2018). When only a minor fraction of these shared protein is localized to peroxisomes, it will be challenging to confirm their peroxisomal localization by means of immunofluorescence analysis or fluorescent tags, as the peroxisomal staining may be masked by the staining of the other organelles.

Our method described here based on the self-assembling split sfGFP assay provides a sensitive approach to localize and study the topology of such putative peroxisomal membrane proteins in cellulo. We show that coexpression of GFP11-tagged peroxisomal membrane proteins with GFP(1–10)-PTS1 can be used to verify the peroxisomal localization of membrane proteins, while coexpression of the GFP11-tagged proteins with GFP(1–10) can be used to exclude or confirm that the GFP11 tag of the peroxisomal membrane protein is exposed to the cytosolic side of the peroxisomal membrane. Unfortunately, although the self-assembling split sfGFP method can be used very well to study the fate of peroxisomal membrane proteins, it is less suited for soluble proteins due to the previously described piggybacking phenomenon, as a consequence of which non-PTS-containing soluble proteins can be coimported with PTS-containing proteins, provided that they can interact in the cytosol prior to the import.

To demonstrate the value of the self-assembling split sfGFP method, we studied the location and orientation of four acyl-CoA synthetases previously reported to be partially located in peroxisomes. The acyl-CoA synthetases have distinct, but overlapping, substrate specificities: SLC27A4 has substrate specificity for saturated long-chain (C16:0) and very long-chain (C24:0) fatty acids (Herrmann et al. 2001; Hall et al. 2005; Jia et al. 2007); SLC27A2 for long-chain and very long-chain, saturated and polyunsaturated fatty acids (Melton et al. 2011) but is also able to activate branched-chain fatty acids, including phytanic and pristanic fatty acids (Steinberg et al. 1999); ACSL1 for a wide range of saturated and some monounsaturated long-chain fatty acids (Iijima et al. 1996; Golej et al. 2011); and ACSL4 for polyunsaturated fatty acids (C20:4, C20:5, and C22:6) (Golej et al. 2011; Shimbara-Matsubayashi et al. 2019). When considering the different substrate affinities, our finding that the acyl-CoA synthetase domains of peroxisome-bound SLC27A2 and SLC27A4 are facing the peroxisomal lumen suggests that (1) SLC27A2 and SLC27A4 may be involved in the reactivation of very long-chain fatty acids and SLC27A2 of polyunsaturated and branched-chain fatty acids after their import into peroxisomes by ABCD transporters, (2) SLC27A2 may be involved in the activation of intraperoxisomally-generated pristanic acid, and (3) SLC27A2 and SLC27A4 both can activate the long-chain fatty acids released by the GNPAT during the de novo ether lipid synthesis. Our finding that the acyl-CoA synthetase domain of peroxisome-bound ACSL1 is facing the cytosol suggests that this synthetase may be involved in activating long-chain fatty acids prior to their import into peroxisomes by the ABCD transporters. This finding is also in line with the reported observation that ACSL1 interacts with ACBD5 (Young et al. 2018), a peroxisomal membrane protein that is also facing the cytosol (Costello et al. 2017).

We could not confirm the previously reported peroxisomal localization of ACSL4, which was based solely on coenrichment of the protein with peroxisome-enriched rat liver subcellular fractions (Lewin et al. 2002). In line with our results, ACSL4 was also not identified in proteomic studies of peroxisome-enriched mammalian subcellular fractions (Wiese et al. 2007; Gronemeyer et al. 2013) or after overexpression of GFP- or FLAG-tagged variants of ACSL4 in COS cells (Küch et al. 2014).

In conclusion, we developed a sensitive approach, which allows for demonstration of the peroxisomal localization and topology of membrane proteins in cellulo. The approach will in particular assist in identifying membrane proteins that partly colocalize to peroxisomes.

### Supplementary Information

Below is the link to the electronic supplementary material.

Supplementary file1Figure S1 a After coexpression with GFP(1–10)-PTS1, GPI-GFP11 is colocalized with peroxisomal marker protein ACBD5. Immunofluorescence analysis of HeLa cells transfected with a plasmid encoding GFP(1–10) and GPI-GFP11 using anti-ACBD5 antibodies (Alexa594, red), sfGFP signal appears as green, DAPI signal appears as blue; image at the right side presented as overlay of sfGFP, Alexa594 and DAPI. b-f SLC27A2 and SLC27A4 are localized to ER and ACSL1 is localized to mitochondria. Live-cell imaging of HeLa cells cotransfected with plasmids encoding GFP(1–10) and (b, d) SLC27A2-GFP11 or (c, e) SLC27A4-GFP11 or (f) ACSL1-GFP11. Cells were incubated with (b-c) ER-Tracker or (d-f) Mito-Tracker. sfGFP signal appears as green, ER-Tracker and Mito-Tracker signal as red. Images at the right side represent overlays of sfGFP and ER-Tracker or Mito-Tracker (TIF 92760 KB)

Supplementary file2 (DOCX 17 KB)
