# Supplementary material for: Studying the topology of peroxisomal acyl-CoA synthetases using self-assembling split sfGFP
Source: Histochem Cell Biol. 2024 Jan 19;161(2):133–44. doi: 10.1007/s00418-023-02257-7 (PMC10822792; doi:10.1007/s00418-023-02257-7)
Supplement: Supplementary file 2 — Supplementary file2 (DOCX 17 KB) [file 418_2023_2257_MOESM2_ESM.docx]

**Table 1.** List of DNA sequences of oligonucleotides and open reading frames used in this study.

| **Cloning/construct** | **DNA sequence description** | **Oligonucleotide sequence** | **Open reading frames** **sequence** |
| --- | --- | --- | --- |
| Clone *GFP(1-10)* into pcDNA5/frt plasmid to generate GFP(1-10)-pcDNA5/frt plasmid | *Kpn*I-*GFP(1-10)-BamH*I (fw) | TTAATGGTACCATGTCCAAAGGAGAAGAACTG |  |
|  | *Kpn*I*-GFP(1-10)-BamH*I (rev) | TATATGGATCCACTTCCGCCGCCACCTGT |  |
| Clone *linker-PTS1 (from ACOX3)* into GFP(1-10)-pcDNA5/frt plasmid to generate GFP(1-10)-PTS1-pcDNA5/frt | *BamHI-linker-PTS1(ACOX3)-Not*I (fw) | GATCCGGTTCCGGAGGTGAAAACAAACCTGTCATAGGAAGTCTGAAATCGAAGCTCTAGAGC |  |
|  | *BamHI-linker-PTS1(ACOX3)-Not*I (rev) | GGCCGCTCTAGAGCTTCGATTTCAGACTTCCTATGACAGGTTTGTTTTCACCTCCGGAACCG |  |
| Clone linker into pcDNA3 plasmid to generate N-GFP11-pcDNA3 plasmid | *Xba*I-short linker-*GFP11-*stop codon(GGGRDHMVLHEYVNAAGIT*)-*Apa*I (fw) | CTAGAGGTGGCGGCCGTGACCACATGGTCCTTCATGAGTATGTAAATGCTGCTGGGATTACATGAGGGCC |  |
|  | *Xba*I-short linker-*GFP11-*stop codon(GGGRDHMVLHEYVNAAGIT*)-*Apa*I (rev) | CTCATGTAATCCCAGCAGCATTTACATACTCATGAAGGACCATGTGGTCACGGCCGCCACCT |  |
| Clone linker into pcDNA3 plasmid to generate GFP11-C-pcDNA3 plasmid | *Hind*III*-*ATG*-GFP11*-short linker (MRDHMVLHEYVNAAGITGGG)-*Kpn*I (fw) | AGCTTATGCGTGACCACATGGTCCTTCATGAGTATGTAAATGCTGCTGGGATTACAGGTGGCGGCGGTAC |  |
|  | *Hind*III*-*ATG*-GFP11*-short linker (MRDHMVLHEYVNAAGITGGG)-*Kpn*I (rev) | CGCCGCCACCTGTAATCCCAGCAGCATTTACATACTCATGAAGGACCATGTGGTCACGCATA |  |
| *SLC25A17* (without stop codon)*-GFP11*-stop codon |  |  | GGTACCATGGCTTCCGTGCTGTCCTACGAAAGCCTGGTCCACGCCGTGGCCGGAGCCGTGGGAAGCGTGACAGCAATGACAGTGTTTTTTCCCCTGGATACAGCTAGACTTCGACTTCAGGTTGATGAGAAAAGAAAATCCAAAACTACACACATGGTGCTCCTGGAGATCATTAAAGAAGAAGGACTCCTGGCACCATATCGAGGGTGGTTTCCAGTGATTTCCAGTCTCTGCTGCTCCAATTTTGTCTATTTCTACACTTTTAATAGCCTCAAAGCACTCTGGGTCAAAGGTCAACATTCTACCACTGGAAAAGATCTGGTAGTTGGGTTTGTTGCAGGAGTGGTTAATGTGTTGCTAACAACTCCACTCTGGGTGGTAAACACCAGACTGAAGCTTCAAGGAGCAAAATTTAGGAATGAAGACATTGTACCAACAAACTACAAAGGTATCATTGATGCTTTTCATCAGATCATTCGCGATGAAGGAATCTCGGCTTTATGGAATGGCACATTTCCCTCATTGCTGTTGGTCTTCAATCCTGCCATCCAGTTCATGTTTTATGAAGGTTTAAAACGGCAGCTTTTAAAGAAACGGATGAAGCTTTCTTCCTTGGATGTGTTCATCATTGGTGCAGTAGCCAAAGCGATTGCCACCACGGTGACCTATCCCCTGCAGACGGTACAGTCAATTCTGAGGTTTGGGCGTCATAGACTAAACCCAGAAAACAGAACATTGGGAAGTCTTCGGAATATTCTCTATCTTCTTCACCAACGAGTAAGACGTTTTGGAATAATGGGACTCTACAAAGGCCTTGAAGCCAAACTGCTGCAGACAGTCCTCACTGCTGCTCTCATGTTCCTTGTTTATGAGAAACTGACAGCTGCCACCTTCACAGTTATGGGGCTGAAGCGTGCAACCAACACGGATCCGGTGGCGGCCGTGACCACATGGTCCTTCATGAGTATGTAAATGCTGCTGGGATTACATGAGCGGCCGC |
| *GFP11-SLC25A17* |  |  | GGTACCATGCGTGACCACATGGTCCTTCATGAGTATGTAAATGCTGCTGGGATTACAGGTGGCGGCGGATCCGCTTCCGTGCTGTCCTACGAAAGCCTGGTCCACGCCGTGGCCGGAGCCGTGGGAAGCGTGACAGCAATGACAGTGTTTTTTCCCCTGGATACAGCTAGACTTCGACTTCAGGTTGATGAGAAAAGAAAATCCAAAACTACACACATGGTGCTCCTGGAGATCATTAAAGAAGAAGGACTCCTGGCACCATATCGAGGGTGGTTTCCAGTGATTTCCAGTCTCTGCTGCTCCAATTTTGTCTATTTCTACACTTTTAATAGCCTCAAAGCACTCTGGGTCAAAGGTCAACATTCTACCACTGGAAAAGATCTGGTAGTTGGGTTTGTTGCAGGAGTGGTTAATGTGTTGCTAACAACTCCACTCTGGGTGGTAAACACCAGACTGAAGCTTCAAGGAGCAAAATTTAGGAATGAAGACATTGTACCAACAAACTACAAAGGTATCATTGATGCTTTTCATCAGATCATTCGCGATGAAGGAATCTCGGCTTTATGGAATGGCACATTTCCCTCATTGCTGTTGGTCTTCAATCCTGCCATCCAGTTCATGTTTTATGAAGGTTTAAAACGGCAGCTTTTAAAGAAACGGATGAAGCTTTCTTCCTTGGATGTGTTCATCATTGGTGCAGTAGCCAAAGCGATTGCCACCACGGTGACCTATCCCCTGCAGACGGTACAGTCAATTCTGAGGTTTGGGCGTCATAGACTAAACCCAGAAAACAGAACATTGGGAAGTCTTCGGAATATTCTCTATCTTCTTCACCAACGAGTAAGACGTTTTGGAATAATGGGACTCTACAAAGGCCTTGAAGCCAAACTGCTGCAGACAGTCCTCACTGCTGCTCTCATGTTCCTTGTTTATGAGAAACTGACAGCTGCCACCTTCACAGTTATGGGGCTGAAGCGTGCACACCAACACTGAGCGGCCGC |
| Clone *ABCD1 (without stop codon)* into N-GFP11-pcDNA3 plasmid | *Kpn*I-ABCD1-*Xho*I (fw) | TTAATGGTACCATGCCGGTGCTCTCCAGG |  |
|  | *Kpn*I*-ABCD1-Xho*I (rev) | TATATCTCGAGGGTGGAGGCACCCTGGAG |  |
| Clone *ACOX1* into N-GFP11-pcDNA3 plasmid | *Kpn*I-*ACOX1-Xho*I (fw) | TTAATGGTACCATGAACCCGGACCTGCGCAG |  |
|  | *Kpn*I*-ACOX1-Xho*I (rev) | TATATCTCGAGTCAGAGCTTGGACTGCAGTG |  |
| Remove stop codon (using site-directed mutagenesis) from ACOX1-GFP11-pcDNA3 plasmid | DNA region around the stop codon, with the stop codon replaced (fw) | GCAGTCCAAGCTCAGACTCGAGCATGCATCTAG |  |
|  | DNA region around the stop codon, with the stop codon replaced (rev) | CTAGATGCATGCTCGAGTCTGAGCTTGGACTGC |  |
| *Clone ACOX1*(without ATG) into GFP11-C-pcDNA3 plasmid | *Kpn*I*-ACOX1-Xho*I (fw) | TTAATGGTACCAACCCGGACCTGCGCAG |  |
|  | *Kpn*I-*ACOX1-Xho*I (rev) | TATATCTCGAGTCAGAGCTTGGACTGCAGTG |  |
| *GPI* (without stop codon)*-GFP11*-stop codon |  |  | GAATTCATGGTAGCTCTCTGCAGCCTCCAACACCTGGGCTCCAGTGATCCCCGGGCTCTGCCCACCCTCCCCACTGCCACTTCCGGGCAGAGGCCAGCAAAGCGGCGGCGCAAGAGTCCCGCCATGGCCGCTCTCACCCGGGACCCCCAGTTCCAGAAGCTGCAGCAATGGTACCGCGAGCACCGCTCCGAGCTGAACCTGCGCCGCCTCTTCGATGCCAACAAGGACCGCTTCAACCACTTCAGCTTGACCCTCAACACCAACCATGGGCATATCCTGGTGGATTACTCCAAGAACCTGGTGACGGAGGACGTGATGCGGATGCTGGTGGACTTGGCCAAGTCCAGGGGCGTGGAGGCCGCCCGGGAGCGGATGTTCAATGGTGAGAAGATCAACTACACCGAGGGTCGAGCCGTGCTGCACGTGGCTCTGCGGAACCGGTCAAACACACCCATCCTGGTAGACGGCAAGGATGTGATGCCAGAGGTCAACAAGGTTCTGGACAAGATGAAGTCTTTCTGCCAGCGTGTCCGGAGCGGTGACTGGAAGGGGTACACAGGCAAGACCATCACGGACGTCATCAACATTGGCATTGGCGGCTCCGACCTGGGACCCCTCATGGTGACTGAAGCCCTTAAGCCATACTCTTCAGGAGGTCCCCGCGTCTGGTATGTCTCCAACATTGATGGAACTCACATTGCCAAAACCCTGGCCCAGCTGAACCCCGAGTCCTCCCTGTTCATCATTGCCTCCAAGACCTTTACTACCCAGGAGACCATCACGAATGCAGAGACGGCGAAGGAGTGGTTTCTCCAGGCGGCCAAGGATCCTTCTGCAGTGGCGAAGCACTTTGTTGCCCTGTCTACTAACACAACCAAAGTGAAGGAGTTTGGAATTGACCCTCAAAACATGTTCGAGTTCTGGGATTGGGTGGGAGGACGCTACTCGCTGTGGTCGGCCATCGGACTCTCCATTGCCCTGCACGTGGGTTTTGACAACTTCGAGCAGCTGCTCTCGGGGGCTCACTGGATGGACCAGCACTTCCGCACGACGCCCCTGGAGAAGAACGCCCCCGTCTTGCTGGCCCTGCTGGGTATCTGGTACATCAACTGCTTTGGGTGTGAGACACACGCCATGCTGCCCTATGACCAGTACCTGCACCGCTTTGCTGCGTACTTCCAGCAGGGCGACATGGAGTCCAATGGGAAATACATCACCAAATCTGGAACCCGTGTGGACCACCAGACAGGCCCCATTGTGTGGGGGGAGCCAGGGACCAATGGCCAGCATGCTTTTTACCAGCTCATCCACCAAGGCACCAAGATGATACCCTGTGACTTCCTCATCCCGGTCCAGACCCAGCACCCCATACGGAAGGGTCTGCATCACAAGATCCTCCTGGCCAACTTCTTGGCCCAGACAGAGGCCCTGATGAGGGGAAAATCGACGGAGGAGGCCCGAAAGGAGCTCCAGGCTGCGGGCAAGAGTCCAGAGGACCTTGAGAGGCTGCTGCCACATAAGGTCTTTGAAGGAAATCGCCCAACCAACTCTATTGTGTTCACCAAGCTCACACCATTCATGCTTGGAGCCTTGGTCGCCATGTATGAGCACAAGATCTTCGTTCAGGGCATCATCTGGGACATCAACAGCTTTGACCAGTGGGGAGTGGAGCTGGGAAAGCAGCTGGCTAAGAAAATAGAGCCTGAGCTTGATGGCAGTGCTCAAGTGACCTCTCACGACGCTTCTACCAATGGGCTCATCAACTTCATCAAGCAGCAGCGCGAGGCCAGAGTCCAAGGTGGCGGCCGTGACCACATGGTCCTTCATGAGTATGTAAATGCTGCTGGGATTACATGACTCGAG |
| Clone *SLC27A4 (without stop codon)* into N-GFP11-pcDNA3 plasmid | *Kpn*I-*SLC27A4*-*Xho*I (fw) | TTAATGGTACCATGCTGCTTGGAGCCTCTC |  |
|  | *Kpn*I-*SLC27A4-Xho*I (rev) | TATATCTCGAGCAGCTTCTCCTCGCCTGCC |  |
| Clone *SLC27A2 (without stop codon)* into N-GFP11-pcDNA3 plasmid | *Kpn*I-*SLC27A2*-*Xho*I (fw) | TTAATGGTACCATGCTTTCCGCCATCTACAC |  |
|  | *Kpn*I*-SLC27A2-Xho*I (rev) | TATATCTCGAGGAGTTTCAGGGTTTTAGCAC |  |
| Clone *ACSL1 (without stop codon)* into N-GFP11-pcDNA3 plasmid | *EcoR*I-*ACSL1*-XhoI (fw) | TTAATGAATTCATGCAAGCCCATGAGC TGTTC |  |
|  | *EcoR*I*-ACSL1-Xho*I (rev) | TATATCTCGAGAACCTTGATAGTGGAATAGAGG |  |
| Clone *ACSL4 (without stop codon)* into N-GFP11-pcDNA3 plasmid | *Kpn*I-*ACSL4*-XhoI (fw) | TTAATGGTACCATGAAACTTAAGCTAAATGTGCTC |  |
|  | *Kpn*I*-ACSL4-Xho*I (rev) | TATATCTCGAGTTTGCCCCCATACATTCGTTC |  |
